# General Practitioners and Community Pharmacists’ Collaboration in Primary Care: Small Steps for a Major Change

**DOI:** 10.5334/ijic.5612

**Published:** 2021-04-23

**Authors:** Amber Damiaens, Jessica Fraeyman, Sarah Fakroune, Caroline Hutsebaut, Sandrine Roussel, Lieve Van Dyck, Guido Van Hal, Stephan Van den Broucke, Veerle Foulon

**Affiliations:** 1 Department of Pharmaceutical and Pharmacological Sciences, KU Leuven, Herestraat 49, B-3000, Leuven, Belgium; 2 Association of Community Health Centers, Vooruitgangstraat 333 box 10, B-1030 Brussels; 3 Department of Epidemiology and Social Medicine, University of Antwerp, Universiteitsplein 1, B-2610 Wilrijk; 4 Psychological Sciences Research Institute, UCLouvain, Place Cardinal Mercier 10, B-1348, Louvain-La-Neuve, Belgium

**Keywords:** integrated care, primary care, general practice, community pharmacy, interprofessional collaboration

## Abstract

**Background:**

Healthcare authorities worldwide search for ways to develop integrated care and interprofessional collaboration. In Belgium, Medical-Pharmaceutical Concertation (MPC) was introduced as a format to promote constructive dialogues between GPs and community pharmacists (CPs) with a focus on pharmacotherapy.

**Objective:**

To evaluate the implementation of MPC from the perspective of healthcare authorities and GPs/CPs.

**Methods:**

Mixed-methods approach, including semi-structured interviews with stakeholders and service users, observations of MPC meetings and surveys in GPs/CPs. Quantitative data were analyzed using descriptive statistics. Qualitative data were analyzed inductively.

**Results:**

The implementation of MPC took a slow start. Parties involved had divergent views on the goals of the MPC: stakeholders focused on measurable results, while service users aimed on improving interprofessional communication. Additionally, service users felt that the lack of local structures hindered consensus building and implementation of agreements in daily practice. Support from professional associations was considered indispensable for the implementation of MPC. In order to organize this efficiently, the establishment of an independent institution, coordinating the MPC initiative, was highly recommended.

**Conclusion:**

The study confirms that a thorough context assessment prior to implementation of a complex project is needed and that a step-wise approach should be respected to achieve effective interprofessional relationships.

## Introduction

The rising prevalence of chronic diseases and multimorbidity pose a major challenge to modern health care systems [1,2]. For patients with chronic conditions, interdisciplinary health care teams that can deliver integrated care are seen as a way to improve patient experiences as well as health outcomes [3,4]. To achieve such integrated primary care, structured concertation between general practitioners (GPs) and community pharmacists (CPs) is one of the many possibilities. As such, it has become part of clinical practice in several European countries. For instance, in The Netherlands, pharmacotherapy audit meetings (PTAMs) are being regularly organized since 1992 [5]. Likewise, in Switzerland, physicians-pharmacists quality circles (PPQCs) were introduced in 1997 [6]. This form of collaboration in primary care has shown to be effective in the management of multiple chronic conditions, such as hypertension and diabetes type 2. This suggests that it may also be the case for other chronic diseases [7]. Additionally, a systematic review by Kwint et al. showed that the extent of one-on-one collaboration between GPs and CPs is positively associated with the implementation rate of recommendations following a medication review [8].

**Figure 1:**
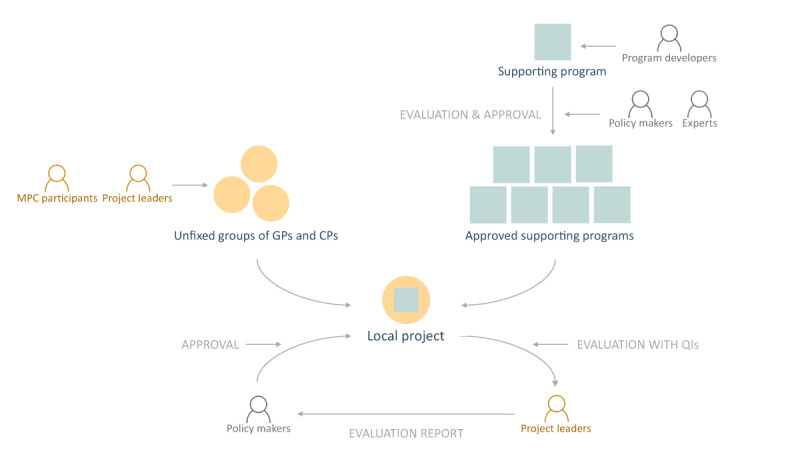
The MPC project.

In Belgium, approximately 16,700 GPs and 21,000 CPs were active in 2019, with an average of 1.5 GPs and 1.8 CPs per 1000 inhabitants [9]. A first official attempt to manage and support structured concertation between these two professional groups was made in 2015. The rules for Medical-Pharmaceutical Concertation (MPC) were set by the publication of a Royal Decree, which aims to facilitate and encourage meetings concerning pharmacotherapy in which GPs and CPs participate in a constructive dialogue. As it is currently implemented, the initiative, which is also referred to as the MPC project, consists of two parts: local projects, and supporting programs (see ***Figure 1***). The local projects are meetings that are held between GPs and CPs on a geographical basis. Each meeting must be organized by at least one GP and one CP, henceforth referred to as the project leaders. The programs provide educational support for the local projects, such as materials and moderators. They are developed by the professional associations for GPs and CPs, universities, educational institutions and scientific organizations. Each program covers a topic related to the rational prescribing, rational dispensing and safe use of medication.

To enhance the implementation of these meetings, the National Institute of Health and Disability Insurance (NIHDI) provides financial support. To receive this funding for the local project, GPs and CPs are required to choose one of the approved supporting programs. In return, the NIHDI expects these meetings to improve quality of care. To capture this, project leaders are asked to evaluate their progress by measuring self-formulated quality indicators (QIs) that reflect the agreements made at the meeting.

The current study aimed to assess and evaluate the local implementation of this government-funded MPC project in Belgium from the perspective of stakeholders and service users (GPs and CPs).

## Methods

### Study population

The study involved two distinct phases. The study population of the first phase consisted of three groups of stakeholders: policy makers, experts and program developers. Policy makers were representatives from the NIHDI. Experts were individuals from health insurance funds or universities. Both groups are involved in the evaluation and approval of submitted programs. Program developers were individuals or representatives of organizations who were qualified to design and submit supporting programs.

For the second phase of the study, service users were included. These were the GPs and CPs who participated in the local MPC and the GPs and CPs who organized the MPC (=project leaders).

### Data collection and analysis

The first phase of the study was performed in 2017. It had a qualitative explorative study design and consisted of semi-structured interviews with policy makers, experts and program developers (‘Study population’). Semi-structured interviews were performed by JF, a social scientist, and SR, a psychologist, both female post-doc researchers, and supported by an interview guide. The interview guide consisted of open-ended questions exploring the role of the stakeholder in MPC, goals, opportunities and threats that could be considered in the further implementation of MPC. Interviews were audio-recorded to facilitate the inductive analysis, supported by the conceptual framework on implementation fidelity (IF), as proposed by Carroll et al. and adapted by Hasson [10,11]. The analysis was done through a series of independently writing summaries of the interviews, discussions and mind-mapping by the two aforementioned researchers. The IF framework allowed to investigate the fidelity with which the MPC project was implemented as planned and which factors influenced this.

The second phase of the study was performed in 2018 and involved a mixed-method study design, which included the analysis of the approved programs and projects, observations of local projects, surveys of service users (collected immediately after the observations) and semi-structured interviews with project leaders (‘Study population’). All three types of data collection were performed by CH, AD, LV and SF (two community pharmacists and two psychologists, respectively). Observations were supported by an observation template, surveys by an online form and semi-structured interviews by an interview guide. These data collection materials were based on the Theory of Planned Behaviour, as well as the theoretical concepts of ‘community engagement’ and ‘community participation’. Quantitative observation and survey data were analysed descriptively in Excel. The analysis of the interviews was facilitated by audio-recording and verbatim transcriptions and was supported by the aforementioned theoretical frameworks [12,13,14,15]. The combination of these theories allowed to identify perceived individual motivational factors, as well as perceived contextual factors that might determine the actual participation in the local MPC. Each interview was independently coded by two members of the research team, which was supported by Excel, and was followed by discussion and solving of discrepancies.

After the analysis of the separate study phases was finalized, with the purpose of integrating the findings of both phases, an inductive approach was used to generate overarching themes. Using different theoretical approaches in separate phases of the study (‘Data collection and analysis’) provided us with an extensive and in-depth view on the implementation of the MPC in Belgium. In this paper, the focus will be on the major findings that are thought to be relevant for an international audience. For that purpose, the results of both phases will be reported in an integrated manner.

### Trustworthiness

Different approaches were applied to ensure trustworthiness of our study findings. Firstly, trustworthiness was supported by the careful documentation of all study procedures and changes therein. Additionally, several forms of triangulation were applied to improve credibility. Both data collection and analysis were performed by a multidisciplinary research team, ensuring investigator triangulation. Method triangulation and data source triangulation were performed by collecting data through interviews, observations and surveys, and by involving the perspectives of all involved parties (e.g. stakeholders and service users), respectively. To further increase trustworthiness, the research teams of both phases regularly held meetings to discuss findings. Likewise, with the purpose of integrating the results of both phases, researchers collaborated closely across teams. Throughout the study, by means of regular multidisciplinary meetings, the potential influence the members of the research team may have had on the study outcomes was limited. Although pharmacists were involved in the study, we believe this had minimal impact on data collection and analysis, since they were 1) not involved in any aspect of the MPC project prior and during the study, and 2) accompanied by other professionals, including psychologists and social scientists, during each step of the way.

### Ethics approval

Since no personal data was collected, but rather the opinion of stakeholders already involved in the MPC project, no approval of the ethics committee was needed for phase one of the study. The second phase of the study was approved by the Ethics Committee for the Social Sciences and Humanities of the University of Antwerp in October 2018.

## Results

### Study population

A total of 22 interviews was performed with stakeholders in phase one of the study. During phase two, the research team performed 15 interviews and 18 observations, and collected 217 surveys from service users. Characteristics of the study population are given in ***Table 1***.

**Table 1:** Characteristics of the study population.


PHASE 1 – STAKEHOLDERS	INTERVIEWS, N	22

	Language, n Dutch French Function, n Policy maker Stakeholder External expert Mean duration, min	13 9 8 10 4 60

**PHASE 2 – SERVICE USERS**	**INTERVIEWS, N**	15*

	Language, n Dutch French Function, n GP CP Mean duration, min	6 9 5 11 55

	**OBSERVATIONS, N**	**18**

	Language, n Dutch French Participants, n GP CP Other** Mean duration, min	15 3 195 181 39 129

	**SURVEYS, N**	**217**

	Language, n Dutch French Function, n GP CP Other** Age, median (IQR)	169 48 102 99 16 41 (27)


* One Dutch interview was performed with one GP and one CP simultaneously. ** Others included nurses, intern pharmacists, medical specialists.

### High expectations overruled

During both phases of the study, it became clear that the expectations about the MPC were tempered by the reality. Firstly, according to several policy makers, the number of approved programs and local projects remained far below the desired number. Although budget was foreseen to support up to 800 local projects per year, by the end of 2018, only 112 local projects had been approved since the launch of the MPC project in 2015. Furthermore, service users indicated that not all 16 supporting programs approved by NIHDI were relevant for daily practice. Additionally, during the interviews, it became clear they were confronted with many barriers with regard to the organization of local projects (see further). Therefore, it could be concluded that the MPC initiative, funded by the Belgian government, has taken a slow start.

### Divergent views on the goal of MPC

One of the issues that arose from the interviews was the interpretation of the goal of MPC, which differed considerably between stakeholders and service users. Although all parties agreed on the long-term goal, i.e., for care to become ‘more efficacious on a regional level’, not everyone agreed on the steps that had to be taken to achieve this goal. Policy makers wanted to see measurable results from the local projects (by means of QIs), while service users preferred to first improve the quality of the collaboration through communication and dialogue.

“The role of MPC in primary health care is to increase dialogue between pharmacists and GPs. What the policy makers want to achieve with MPC is less clear but seems to focus too much on economic savings (with the use of the numeric quality indicators). It seems unproductive to regard this as the primary target of MPC. The primary goal is to tackle the communication problem.” (Phase 1, expert)

“It is not our intention for physicians and pharmacists to just sit together, talk about something and get money for that. We want to standardise MPC and see measurable effects of these efforts.” (Phase 1, policy maker)

“The main goal that we have had in mind from the beginning, is to improve the communication between physicians and pharmacists. That is still the main goal.” (Phase 2, GP 1)

“I know there are indicators for quality improvement. We discussed that last time, but for us, the concept is very, very unclear. We have absolutely no idea what type of indicator we should use, how we should verify our choice, etc.” (Phase 2, CP 8)

### The importance of getting acquainted

**Figure 2:**
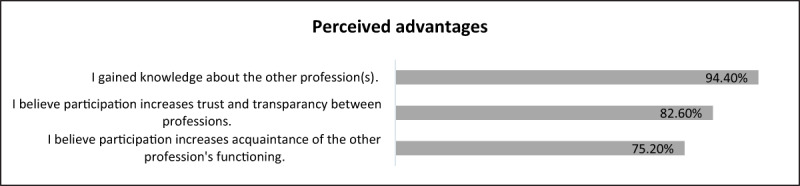
Results of perceived advantages of MPC, % (survey, n = 217).

According to the survey, almost half of the GPs and CPs (45.2%, n = 98/217) indicated that they were participating for the first time in a local project. Additionally, the objectives mentioned most by both parties during the interviews were to get to know each other, get acquainted with the other professional’s way of working and to improve communication between them. Results from the survey confirmed that by participating in the MPC, service users gained knowledge about the other profession, believed participation increased trust between both professions and believed participation helped them get more acquainted with the other profession (see ***Figure 2***). During the interviews, project leaders indicated that a better patient care is what they expected to get out of this.

**Figure 3:**
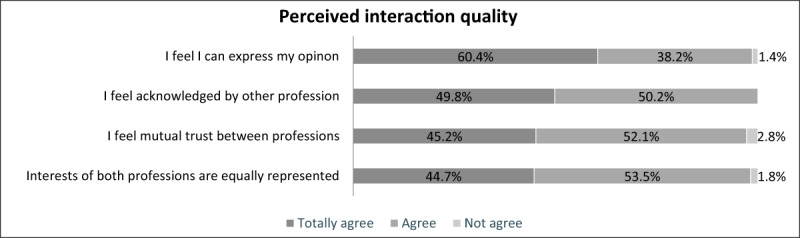
Results of perceived interaction quality during the MPC meeting, % (survey, n = 217).

The atmosphere during the MPC was perceived as good and interaction quality was perceived as spontaneous. According to the survey, the majority of service users felt that they were able to express their opinion and felt mutual trust between the different professional groups. Moreover, all participants felt acknowledged by the other professional group(s) and almost everyone felt like the interests of both professional groups were equally represented (see ***Figure 3***).

In 13 of the 18 observed MPC meetings, there were no indications of one or the other professional group dominating the interaction. However, during the interviews, some CPs expressed feelings of inferiority towards the GPs and feared that the latter would not adapt their daily practice according to the new agreements made at the meetings.

“For me personally, it’s still difficult to consider a physician as my equal. It might be a question of education or behavior, but we’re not yet two [equal] academics, [since] he may have done one or two more years. But no, certainly not superior, inferior.” (Phase 2, CP 2)

### Keeping a local focus gets one motivated

Only in ten of the observed MPC meetings concrete agreements were made. An example of an agreement was to mention either “CKD” (chronic kidney disease) or the eGFR value on the GP’s prescription to inform the pharmacist about the patient’s decreased renal function (of course, upon patient’s consent). In six of these cases, the agreements would be disseminated and communicated among participants. Only in three cases, plans were made about the follow-up of these new agreements. Additionally, interviewed GPs and CPs indicated that agreements made at the meetings were barely put into practice, which resulted in a feeling of failure. However, they acknowledged that making agreements could have been thwarted by the attendance of health care professionals from different regions of the country. Participants felt that the lack of local structures (e.g. fixed groups of GPs and CPs), made it more difficult to make agreements at the meetings and to implement them in daily practice. Therefore, keeping the meetings local was regularly mentioned as a motivating factor for GPs and CPs to attend.

“It’s useless to meet up with a GP from 10 kilometres away, absolutely useless.” (Phase 2, CP 7)

### Getting help from the professional associations

Project leaders found the administrative process to organize a local project unclear and too extensive. They considered the support from professional associations indispensable for the organization of the MPC. These organizations mainly helped with practical arrangement, such as assist in the application procedure to receive funding. Although stakeholders from these organizations agreed that this is part of their job, they indicated that this would not be feasible for an increasing number of future local projects. Therefore, it was suggested by several stakeholders to set up an independent institution to carry all responsibilities with regard to the MPC initiative. This institution would be responsible for the dissemination and implementation of MPC, including the evaluation and approval of both supporting programs and local projects, as well as the provision of clear and accessible information for all parties involved.

“There really is a need for an online forum where programs are available and requests for financial support can easily be submitted, to make the application for a local MPC more accessible for individual physicians and pharmacists.” (Phase 1, program developer)

### Willingness to participate in future MPCs

**Figure 4:**
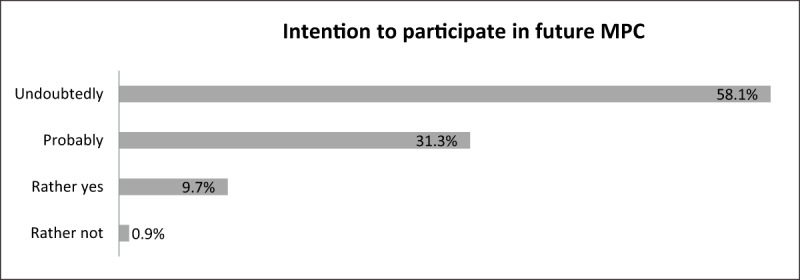
Intention to participate in future MPC meetings, % (survey, n = 217).

As revealed by the survey, most service users had become more enthusiastic about MPC at the end of the meeting than before. Additionally, GPs and CPs indicated that they would certainly or probably participate in future MPC projects (see ***Figure 4***). Likewise, project leaders indicated that they were willing to organize future local projects. Some of them even had specific plans already.

## Discussion

Interdisciplinary health care teams are considered as a way to improve patient experiences and health outcomes. Following the initiatives in several European countries, the Belgian government set out the rules for MPC in 2015 by publishing a new Royal Decree. To enhance the implementation of this Decree, the NIHDI provided financial and educational support. This study was the first to assess the implementation of this government-funded MPC project in Belgium.

### Discussion of major findings

#### The need for context assessment

Inspired by the success of matching initiatives in other European countries (e.g. PTAMs in The Netherlands), the idea of MPC was launched with high expectations regarding its dissemination and effectiveness. Our results show that participant responsiveness, one of the indicators to assess implementation fidelity, remained low and that these expectations were not fully met [10]. The uptake of the initiative was limited, only a small number of local projects had been approved compared to the budget that was made available for their organization, and only a handful of supporting programs was available by the end of the study. Additionally, several impeding factors were identified that need to be addressed for the MPC project to increase. A possible explanation for this lacklustre implementation is the absence of a preceding context assessment. Previous research has shown that the context in which a complex project such as MPC is situated may significantly influence its implementation and effectiveness [16,17,18]. Likewise, the needs nor previous experiences of potential service users with regard to interprofessional GP-CP collaboration were investigated prior or during the development of the MPC project. This may have resulted in a project untailored to their needs and subsequently a lack of their participation [14].

#### The need for a clearly formulated goal of MPC

A common understanding of the operational goals of MPC seems to be lacking. According to the IF framework, this may have been caused by a lack of comprehensiveness of the policy description [10]. This is supported by the Normalization Process Theory (NPT), another widely-known and frequently-used implementation framework, which states that the goal of an initiative should be clear to all parties in order to streamline operations at national, regional and local level and thus to ensure long-term implementation in daily practice [18]. Additionally, this would lead to an increased understanding of the expectations NIHDI has with regard to the measurement of self-formulated QIs.

#### The need for a step-wise approach

Our results show that the MPC is seen as a means to quality improvement through more efficacious care. However, a step-wise approach for implementation is recommended to ensure the quality of delivery, as expected by NIHDI [10]. GPs and CPs indicated that the development of a trusting relationship is the current objective of the MPC project and a means to achieve the long-term goal, i.e. to achieve better patient care. This fits with the collaborative working relationship (CWR) model suggested by McDonough et al., according to which effective CWRs develop in multiple stages and require trust and professional recognition as essential conditions to achieve effective collaborative practice [19,20]. More recently this has been confirmed in the meta-model of physician-CP collaboration (PCPC) proposed by Bardet et al., which states that trust and interdependence including mutual respect and role recognition are core determinants of interprofessional collaboration [21].

Project leaders mentioned that by participating in local projects, they gained knowledge about the other profession and communication improved. They also expressed the same objectives: improve trust and communication and subsequently achieve better patient care. These perceived benefits and shared goals, as well as mutual role recognition, have been previously identified as prerequisites for effective CWRs [22,23,24]. Nonetheless, feelings of inferiority towards the GPs were experienced by a minority of the interviewed CPs. This perceived hierarchy is one of the main barriers to interprofessional care and needs to be addressed to ensure a safe and trusting environment where service users can participate in a constructive manner [15,22]. It can be expected, however, that these feelings will decrease as a result of the improved interprofessional communication. Besides this, pharmacists should be encouraged to use their knowledge and expertise to counteract the existing power balance. Since physicians are the only group of practitioners legally permitted to prescribe medication, the balance currently shifts in their favour. The more balanced the power, the more likely a CWR will be formed [15,19].

#### The need to keep it local

It is thought that after creating trust, service users will be able to make more concrete agreements on the prescribing and dispensing of medication. As our results show, agreements were formulated in a minority of meetings and service users indicated that these were seldom put into practice, which resulted in a feeling of failure. This may result in a lack of perceived effectiveness, another prerequisite to achieve successful collaborative practice [25]. A possible explanation for this is the conflicting interpretation of the goal of MPC. It might seem irrelevant to service users to measure QIs concerning the pharmacotherapy of their patients if their objective is to improve the interprofessional communication. Another explanation for this could be the absence of local structures, such as fixed groups of GPs and CPs. Current guidelines for the organization of a local project contain no information on who should be invited and how many participants should preferably be present during the MPC meeting. Therefore, the possibilities to determine fixed groups of GPs and CPs should be further explored. This could possibly be supported by the formation of the new ‘primary care zones’ in the Flemish part of Belgium and Brussels [26]. As the CWR model suggests, the proximity of practices may have an influence on the working relationship [19]. It has been mentioned in previous studies that working with practitioners in the same geographical area is easier than working with practitioners who work further away [23,27]. Once positive outcomes of the local projects become visible for all parties involved (i.e. GPs, CPs and patients), the trust between practitioners will further increase and the development of CWRs will be enhanced [19].

#### The need for realistic administrative requirements

The administrative procedures for the organization of local projects seem to have hindered the implementation of the MPC project. These procedures were unanimously described as too extensive and too complex by project leaders, which meant they had to ask for help of professional associations. Leaving these procedures untouched might result in a decrease of service users’ perceived behavioural control and eventually in them not organizing local projects anymore [13]. Additionally, to limit the burden on professional associations, the idea of setting up an institution, independent from NIHDI, to take up the responsibility for the MPC project, should be explored as a facilitating strategy [10]. The Dutch Institute for Rational Use of Medicine (Instituut Verantwoord Medicijngebruik, IVM) in The Netherlands could serve as a source of inspiration for this [28]. This organization provides easily accessible information and guidance for Dutch GPs and CPs with regard to the organization and evaluation of PTAMs.

### Future perspectives

The MPC initiative is appreciated by stakeholders and service users and enthusiasm seems to be growing. To further enhance the uptake of MPC, the implementation process should be structured and supported by NIHDI or by an independent institution as suggested. Additionally, aspects that are currently hindering the process should be addressed. With these conditions in mind, the launch of MPC can be seen as the kick-off to the formation of CWRs between GPs and CPs in Belgium. Yet the MPC initiative is only the first step in delivering excellent integrated care. In several countries, more advanced formats of GP-CP collaboration are already in place. *Practice pharmacists*, for example, are active within general practices in Australia, USA and UK to perform tasks such as patient education and counselling, as well as medication reviews [29]. Another example is that of the *prescribing pharmacist* in the UK. Pharmacist supplementary prescribing has been on the agenda since 2003, followed by pharmacist independent prescribing in 2006 [30]. The latter allows trained pharmacists to prescribe within their competence the same medications as physicians with the aim to improve patient outcomes, patient access to medications, patient choice, as well as to improve team work. Based on our results, we can state that a strategic approach will be necessary if progress towards such advanced interprofessional practices is aimed for.

### Strengths and limitations

This study is not without its limitations. An important limitation is that no data was collected of GPs and CPs who are currently not participating in local MPC. Including their perspective could have identified additional barriers to participation. Likewise, it should be considered that only motivated stakeholders and service users were included in the study.

However, despite these limitations this study has several strengths. It was the first study to assess the implementation of the MPC project in Belgium after the publication of the Royal Decree in 2015. Additionally, the use of a mixed-methods approach involving both quantitative and qualitative data allowed for a more profound understanding of the implementation process.

## Conclusion

This study confirms that a thorough context assessment should be performed prior to implementation of a complex project. Failing to do so may have been an important reason for the low uptake of the MPC in Belgium so far. Additionally, the results show that a step-wise approach is needed to achieve effective CWRs. In a first step, what might be supported by the creation of fixed groups, a foundation of trust and mutual respect should be obtained between GPs and CPs. Subsequently, these groups need to build up their experience with regard to making concrete agreements with the aim of improving rational prescribing, rational dispensing or safe use of medication. Based on this experience, they can move towards the delivery of interprofessional care of which the impact can be assessed by measuring QIs, either self-developed by these local groups or defined by the program developers. Provided that these different steps of implementation are respected and the identified barriers to implementation are tackled, uptake of the MPC is expected to increase since enthusiasm is growing among all parties involved.
